# Aldose reductase inhibition decelerates optic nerve degeneration by alleviating retinal microglia activation

**DOI:** 10.1038/s41598-023-32702-5

**Published:** 2023-04-05

**Authors:** Mishal Rao, Yu-Kai Huang, Chia-Chun Liu, Chandler Meadows, Hui-Chun Cheng, Mengli Zhou, Yu-Chih Chen, Xin Xia, Jeffrey L. Goldberg, Andrew M. Williams, Takaaki Kuwajima, Kun-Che Chang

**Affiliations:** 1 Department of Ophthalmology, Louis J. Fox Center for Vision Restoration, University of Pittsburgh School of Medicine, 203 Lothrop, Pittsburgh, PA 15213 USA; 2 Department of Neurobiology, Center of Neuroscience, University of Pittsburgh School of Medicine, Pittsburgh, PA 15213 USA; 3 Graduate Institute of Medicine, College of Medicine, Kaohsiung Medical University, Kaohsiung, 80708 Taiwan; 4 Division of Neurosurgery, Department of Surgery, Kaohsiung Medical University Hospital, Kaohsiung, 80708 Taiwan; 5 Department of Surgery, Kaohsiung Municipal Ta-Tung Hospital, Kaohsiung, 80145 Taiwan; 6 Department of Computational and Systems Biology, Hillman Cancer Institute, University of Pittsburgh School of Medicine, Pittsburgh, PA 15260 USA; 7 Spencer Center for Vision Research, Byers Eye Institute, School of Medicine, Stanford University, Palo Alto, CA 94304 USA

**Keywords:** Neuroscience, Diseases

## Abstract

As part of the central nervous system (CNS), retinal ganglion cells (RGCs) and their axons are the only neurons in the retina that transmit visual signals from the eye to the brain via the optic nerve (ON). Unfortunately, they do not regenerate upon injury in mammals. In ON trauma, retinal microglia (RMG) become activated, inducing inflammatory responses and resulting in axon degeneration and RGC loss. Since aldose reductase (AR) is an inflammatory response mediator highly expressed in RMG, we investigated if pharmacological inhibition of AR can attenuate ocular inflammation and thereby promote RGC survival and axon regeneration after ON crush (ONC). In vitro, we discovered that Sorbinil, an AR inhibitor, attenuates BV2 microglia activation and migration in the lipopolysaccharide (LPS) and monocyte chemoattractant protein-1 (MCP-1) treatments. In vivo, Sorbinil suppressed ONC-induced Iba1 + microglia/macrophage infiltration in the retina and ON and promoted RGC survival. Moreover, Sorbinil restored RGC function and delayed axon degeneration one week after ONC. RNA sequencing data revealed that Sorbinil protects the retina from ONC-induced degeneration by suppressing inflammatory signaling. In summary, we report the first study demonstrating that AR inhibition transiently protects RGC and axon from degeneration, providing a potential therapeutic strategy for optic neuropathies.

## Introduction

Central nervous system (CNS) axons and neurons are incapable of spontaneously regenerating in mammals^1^. Injury or insult to CNS axons causes irreversible damage leading to neurological and neurodegenerative disorders^2^. The optic nerve (ON) is a sensory nerve critical for vision. The ON transmits signal from the eye to the brain through axons of retinal ganglion cells (RGCs), which reside in the nerve fiber layer of the neural retina. Unmyelinated RGC axons exit the eyes globe at the ON head (optic nerve head). Posterior to the ONH, the axons are myelinated by supporting oligodendrocytes, a type of glial cell. Given their delicate structure, ON axons are vulnerable to insult.

Unlike other vertebrates^3^, mammals cannot spontaneously regenerate damaged axons of RGC after ON injury due to various inhibitory factors^4,5^. Since RGCs are critical for vision, knowing what other cells influence RGCs and RGC axon survival, especially when injured, is essential. Other than neuronal cells, the mammalian retina contains three types of glial cells: Müller glia (MG), astrocyte glia (ASG), and retinal microglia (RMG)^6^. ASG cell bodies and processes are found in the nerve fiber layer and closely interact with RGCs and their axons in the ONH^7^. RMG, the primary immune cells in the retina that respond to stress, are found at varying densities in every retinal layer, including the ganglion cell layer (GCL), where they interact with RGCs^7^. Since the eye is immune-privileged, it adapts RMG to act as innate immune cells in response to injury^8^. In optic neuropathies, axons of RGCs are injured, which leads to progressive RGC loss and neurodegeneration. In response to optic neuropathies, ​RMG adapt to their surrounding environment morphologically and switch from a “resting state” to an “activated state.” Accumulating evidence shows that activated RMG thereby function as innate immune cells in response to injury and have been associated with RGC pathology^9–11^.

Various approaches are being investigated to promote RGC survival and RGC regeneration, including gene^5,12–15^ and stem cell replacement therapies^16–19^. Using the optic nerve crush (ONC) model, we induce acute axonal trauma to investigate the underlying mechanisms of RGC survival and axon regeneration^5,20^. ONC leads to axonal degeneration towards the proximal cell body and the distal axons in conjunction with inflammatory cytokine production, causing RGC death^21^. Thus, prevention of retinal inflammation could be a therapeutic strategy for alleviating RGC and axon degeneration after ON injury.

Aldose reductase (AR, AKR1B1) is the first enzyme in the two-step polyol pathway that converts glucose to sorbitol in an NADPH-dependent manner^22,23^. Hyperglycemia acts as a noxious stimulus and increases AR polyol pathway signaling^24^, which leads to the production of reactive oxygen species (ROS), accumulation of advanced glycation end-products (AGE), and macrophage/microglia activation^25–27^. In addition to its response to hyperglycemia, AR has also been reported to be involved in other inflammatory diseases like diabetes, cancer, sepsis, asthma, etc.^28–30^. An increase in the AR polyol pathway enhances the production of cytokines/chemokines and ROS, leading to the NADH/NAD + redox imbalance in various inflammatory diseases^29,31–33^. As ocular inflammation is a direct feature of ocular neuropathies, modulating the immune response is vital to promote axonal growth^1,34^. Under normoglycemic conditions, AR inhibition suppresses inflammatory signaling via an alternative mechanism. The AR polyol pathway leads to the decrease of NADH/NAD + redox balance which results in the upregulation of apoptosis and Sirt1-mediated inflammation, thereby promoting ROS production^28,35,36^. Inhibition of the AR polyol pathway suppresses inflammatory response within the cell and provides a regulatory mechanism of AR in microglial activation. Furthermore, AR has been linked to ocular inflammation via the NF-κB pathway, and previous studies show that AR inhibition might provide a role in regulating ocular inflammation^30,37^. In the retina, AR has been observed in RMG^38^, suggesting that RMG could be a pharmaceutical target against ocular inflammation. Our previous studies reported that the blockade of AR attenuates ocular inflammation and diabetic complications by suppressing RMG activation^9,38^. In this study, we further investigated whether pharmacological inhibition of AR can attenuate ocular inflammation and promote RGC survival and axon regeneration after ON injury. Our data showed that Sorbinil treatment attenuates RMG activation, subsequently promotes RGC survival and delays axon degeneration one week after ONC. Findings from our study can be applied as a therapeutic strategy in translational therapy for optic neuropathies.

## Results

### AR inhibition reduces LPS-induced inflammation and microglia migration

**Figure 1:**
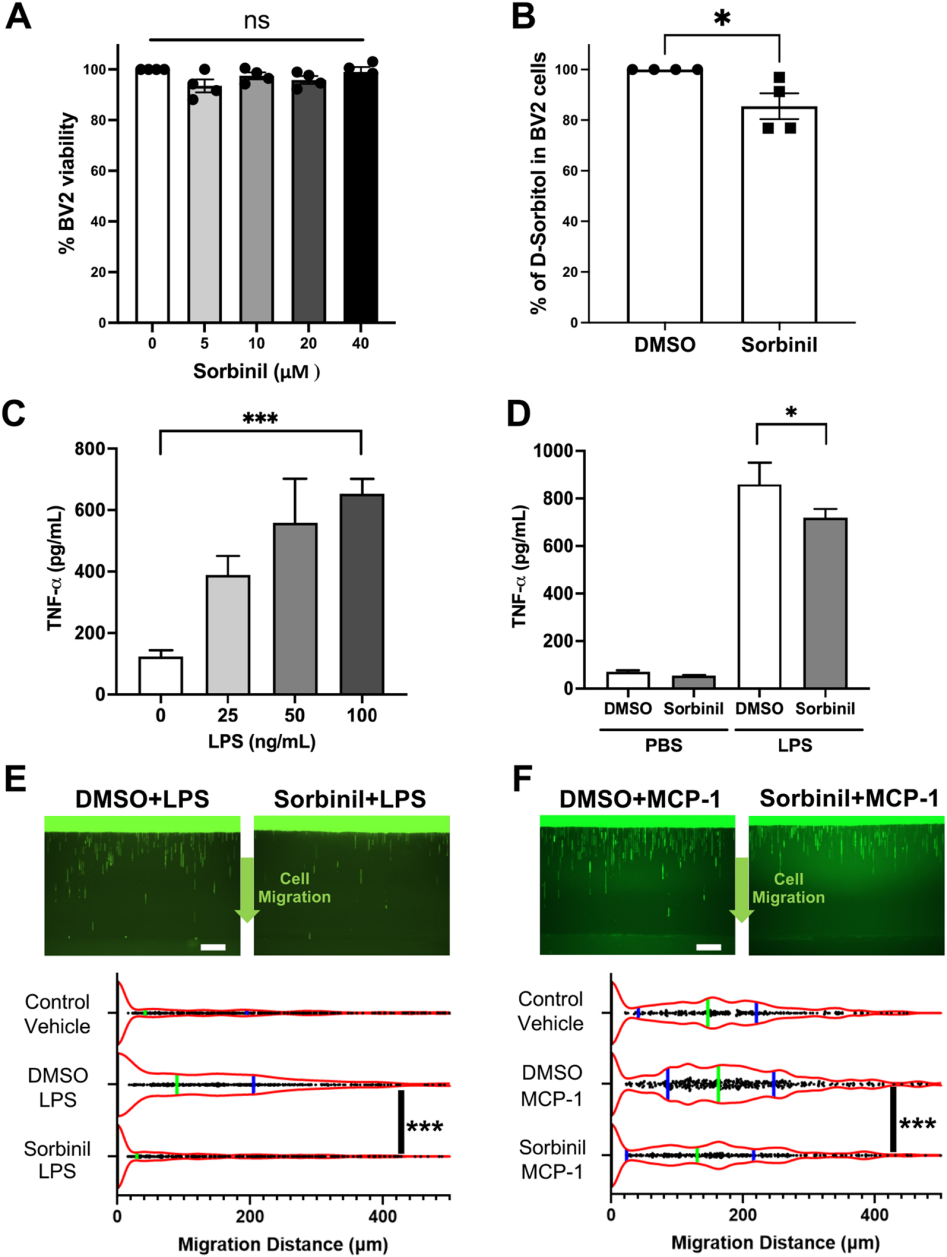
Sorbinil reduces LPS-induced cytokine expression and cell migration in BV2 microglia. **(A)** Sorbinil does not affect cell viability in BV2 microglia as measured by MTT assay. **(B)** Sorbinil treatment attenuates aldose reductase (AR) activity in BV2 microglia, as indicated by the decrease in D-sorbitol concentration. **(C)** LPS induces TNF-α secretion in a dose-dependent manner in BV2 microglia.** (D)** BV2 microglia treated with Sorbinil (20 μM) exhibit reduced TNF-α secretion in response to LPS (100 ng/mL) exposure, compared to vehicle-treated cells. BV2 microglia were pre-treated with Sorbinil (20 μM) for 1 h and then treated with LPS (100 ng/mL) or MCP-1 (30 ng/mL) N ≥ 3 per group. **(E)** Sorbinil treatment inhibits LPS-induced cell migration of microglia cells. Each dot represents the cell migration distance in a channel. The green bar represents the median, and the blue bars represent the quartiles. (n = 500–600 channels). (Scale bar = 200 µm) **(F)** Sorbinil treatment inhibits the chemoattraction of microglial cells induced by MCP-1. Each dot represents the cell migration distance in a channel. The green bar represents the median, and the blue bars represent the quartiles. (n = 300–400 channels). (Scale bar = 200 µm). Statistical significance was determined using Student’s *t-test* (***P < 0.001).

The effect of Sorbinil’s toxicity on BV2 microglia was determined by MTT assay (Fig. 1A). Cell viability was unaffected upon the addition of increasing Sorbinil concentrations. Further, we confirmed that Sorbinil attenuates AR activity by measuring D-Sorbitol levels in BV2 cells (Fig. 1B). Sorbitol can be produced either from AR’s catalysis of glucose via the polyol pathway or from fructose via sorbitol dehydrogenase. The modest reduction of sorbitol can be the result of either Sorbinil incompletely inhibiting the polyol pathway or sorbitol dehydrogenase-mediated reduction of fructose back-filling sorbitol level.

To confirm the role of AR in microglia activation, we treated RMG with lipopolysaccharide (LPS) in the presence of Sorbinil. We found that TNF-α secretion was induced by LPS in a dose-dependent manner (Fig. 1C) and suppressed by Sorbinil (Fig. 1D). Furthermore, because RMG activation is associated with cell motility^39^, we performed RMG migration assay to evaluate the effect of Sorbinil on RMG when exposed to LPS or monocyte chemoattractant protein-1 (MCP-1), a chemokine for the macrophage/microglia recruitment^40^. Data showed that Sorbinil treatment reduces LPS-induced random RMG migration (Fig. 1E). Similarly, Sorbinil prevented the migration of RMG toward MCP-1 (Fig. 1F). These data are consistent with previous studies showing the inhibitory effect of Sorbinil on the RMG migration^38^.

### AR inhibition reduces RMG distribution and inflammation in vivo

**Figure 2:**
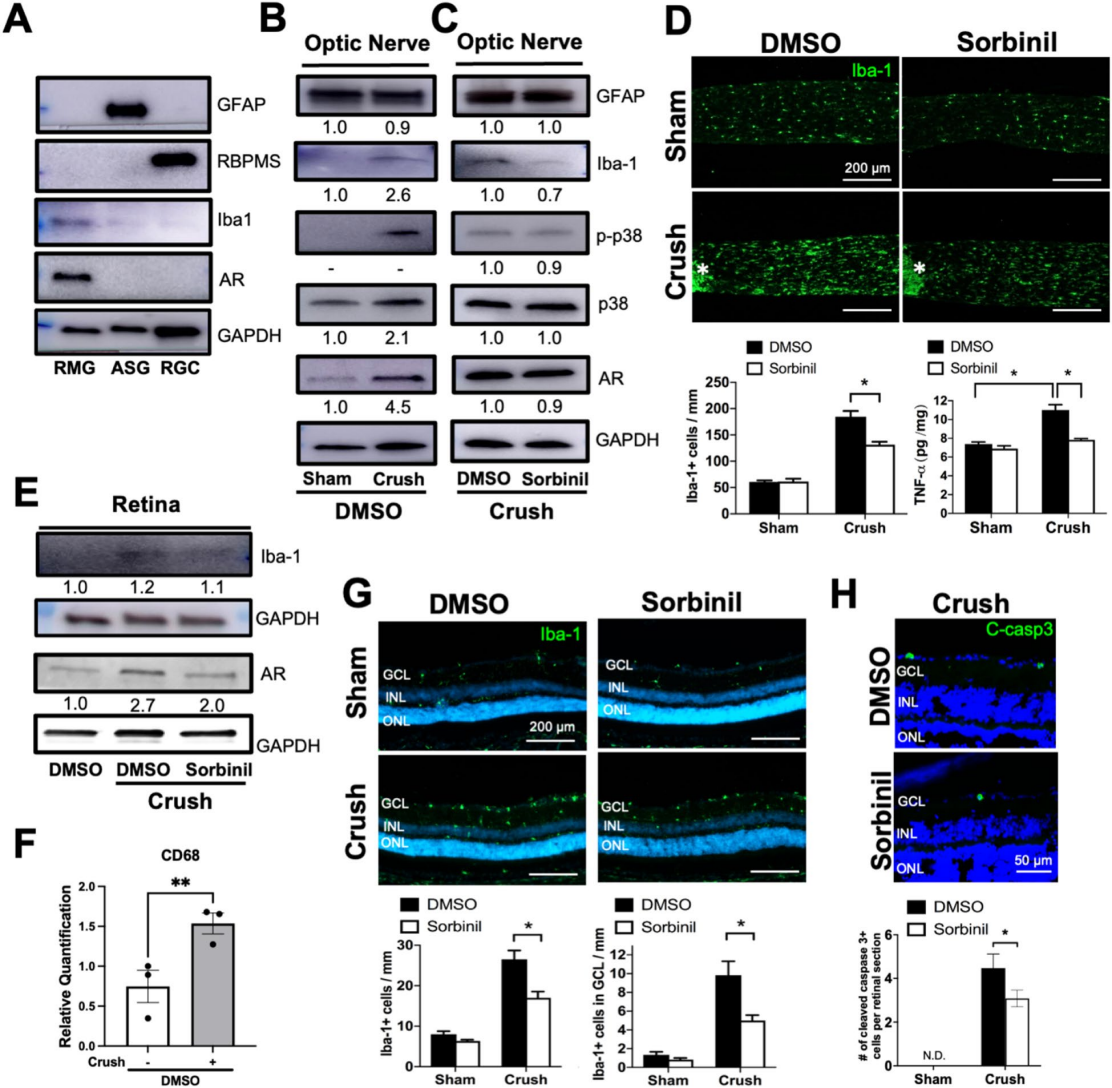
Sorbinil alleviates RMG activation in the retina and optic nerve after ONC. **(A)** AR is highly expressed in RMG compared to ASG and RGC. **(B)** ONC increases AR and Iba1 protein expression in the ON compared to noncrush (sham) control group. Sorbinil treatment reduces ONC-induced AR and Iba1 protein expression in the ON **(C)** and retina** (E).** Sorbinil treatment attenuates RMG distribution and TNF-α secretion in the ON **(D)** and retina **(F).** Asterisk in **(D)** marks the crush site. **(G)** ONC triggers microglial activation as measured by *CD68* gene expression **(H)** Sorbinil treatment reduces the number of cleaved caspase-3 positive cells in the ganglion cell layer (GCL) of the ONC retina, indicating reduced apoptotic cell death. N.D. refers to no detection of Cleaved caspase-3 signal. (N ≥ 3 per group. Statistical significance was determined using one-way ANOVA and post hoc t-test with Tukey correction (*P < 0.05). Mean ± SEM is shown.).

To further examine the effect of AR in vivo, we first evaluated the AR expression pattern in RGC, RMG, and ASG by western blot. In comparison to RGC and ASG, RMG highly express AR (Fig. 2A). Since AR expression was observed primarily in RMG, we next investigated whether AR inhibition prevents RMG activation in the ON and retina. Iba1 (Ionized calcium-binding adaptor molecule-1- a marker of activated microglia/macrophage) expression was elevated in both ONs and retinas (Fig. 2B,C, and E) one week after ONC. ONC increases the number of microglia/ macrophages due to both local microglia cell proliferation and circulating monocyte infiltration^41^. AR inhibitor Sorbinil attenuated Iba1 expression in the crushed ONs and retinas (Figs. 2C and E). As AR is primarily present in RMG, it suggests that the increase of AR expression might be due to RMG distribution in crushed ON and retinas (Fig. 2B, C, and E). Iba1 and AR expression were downregulated in the crushed ON after Sorbinil treatment (Fig. 2C). In addition, glial fibrillary acidic protein (GFAP, marker for activated ASG) expression was not affected after ONC or suppressed by Sorbinil treatment (Fig. 2B,C). Mitogen-activated protein (MAP) kinase p38 is activated in response to environmental stress and has been associated with RGC injury and pathology^42^. We observed that p38 was phosphorylated after ONC, Sorbinil slightly attenuated its phosphorylation in the ON (Fig. 2B,C), suggesting the p38 signaling pathway as a possible AR mediator for microglia activation. Additionally, we confirm that ONC triggers RMG activation by measuring CD68 gene expression in the retina (Fig. 2F). As RMG are known to infiltrate into the injured tissue area, we further investigated the RMG distribution in the retina and ON by immunostaining. In the ON, we observed that the number of Iba1 + cells and TNF-α levels were significantly elevated after ONC and suppressed by Sorbinil treatment (Fig. 2D). In the retina, the number of Iba1 + microglia/macrophage were induced in both GCL and nuclear layers after ONC and attenuated by Sorbinil treatment (Fig. 2G).

We next investigated if ONC would cause apoptosis in the retina. We found cleaved-caspase-3 positive cells in the GCL after ONC, indicating the apoptotic cells (Fig. 2H). The number of apoptotic cells in the GCL per retina section was reduced by Sorbinil treatment, suggesting that AR inhibition alleviates apoptosis in RGC and might be due to the suppression of RMG activation. This is the first study showing the effects of AR inhibition on RMG activation and ocular inflammation in the retina of an ONC model. Administration of Sorbinil through intraperitoneal (IP) injection showed the suppressive effects of RMC activation in the ONs and retinas, which indicates the good permeability of Sorbinil in vivo^38^. We next investigated if AR inhibition affects RGC survival and axon degeneration.

### AR inhibition reduces RGC loss, improves RGC function, and delays axon degeneration

**Figure 3:**
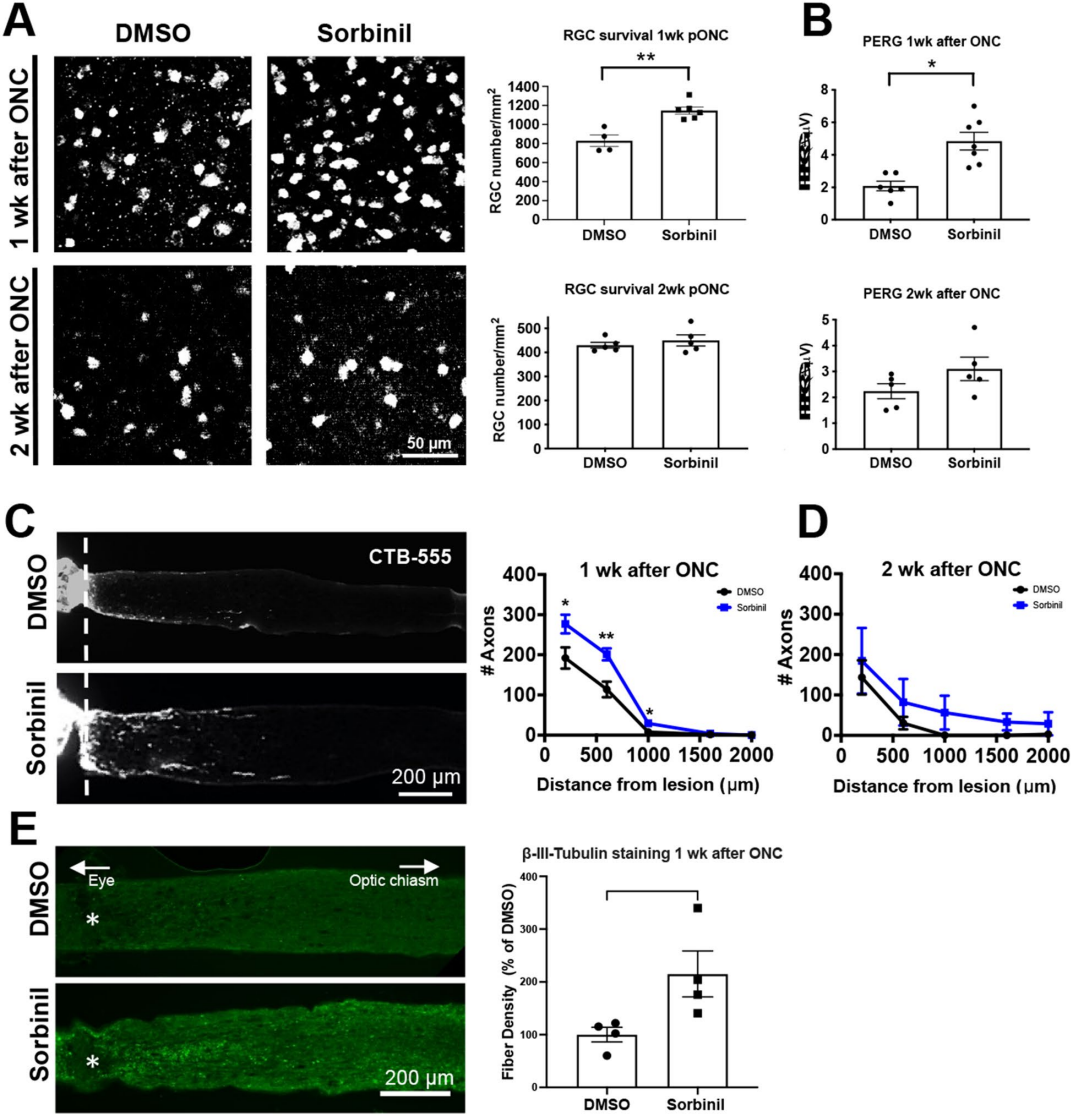
Sorbinil promotes RGC survival, RGC function and delays RGC degeneration one week after ONC. **(A)** Flat-mount retinas were immunostained against RGC-specific marker RBPMS. Sorbinil treatment significantly improves RGC survival one week after ONC, but not two weeks after ONC. **(B)** RGC activity was measured by pattern electroretinography (pERG) in adult retinas. Sorbinil treatment significantly improves pERG one week after ONC. Regenerating axons were visualized by CTB-555 injection two days before euthanasia. **(C)** Sorbinil treatment significantly increases CTB-555 labeled axons one week after ONC. **(D)** Sorbinil does not promote ON regeneration two weeks after ONC. **(E)** Sorbinil treatment significantly delays ON degeneration one week after ONC, as measured by the abundance of βIII-Tubulin staining protein expression. The dashed line in **(C)** and asterisk in **(E)** mark the site of ONC. (**A**, **B, C,** N > 6 eyes per group; **D**, N = 5 eyes per group; **E,** N = 4 eyes per group. Statistical significance was determined using one-way ANOVA and post hoc t-test with Tukey correction (*P < 0.05, **P < 0.01). Mean ± SEM is shown.

Since AR inhibition can attenuate RMG distribution and inflammation after ONC, first, we investigated if AR inhibition also affected RGC survival after ONC. We conducted RGC survival observation one and two weeks after ONC to determine if Sorbinil treatment delays or inhibits RGC death, respectively. RBPMS + RGCs were quantified in the flat mount retina of the crushed control and treatment group. Data showed that Sorbinil treatment did not improve RGC survival two weeks after ONC (Fig. 3A). Next, we wondered whether AR inhibition could alleviate RGC loss in a short-term manner, i.e., if AR inhibition can delay RGC loss one week after ONC. Excitedly, Sorbinil treatment did maintain more RGCs one week after ONC compared to the control (Fig. 3A), suggesting that AR inhibition can transiently protect RGCs from death. In addition, Sorbinil also improved the RGC function one week after ONC by using PERG measurement (Fig. 3B). Next, we investigated the effect of AR inhibition on axon regeneration. Similar to RGC survival, Sorbinil treatment did not improve axon regeneration two weeks after ONC (Fig. 3D) but delayed axon degeneration one week after ONC as measured by βIII-Tubulin protein expression in axons and quantifying CTB-555 expression. (Figs. 3C,E). βIII-Tubulin labeling of axons is a widely established method to determine axonal integrity by measuring the abundance of protein within the axons^43^. One week after ONC, at 500 μm distal from the crush, the density of βIII-Tubulin in Sorbinil treated mice compared to DMSO control is significantly higher, suggesting delayed degeneration (Fig. 3E). These data indicate AR as a therapeutic target to delay the progression of RGC loss and optic neuropathy.

### AR inhibition regulates inflammation related signaling pathways in the ONC retina

**Figure 4:**
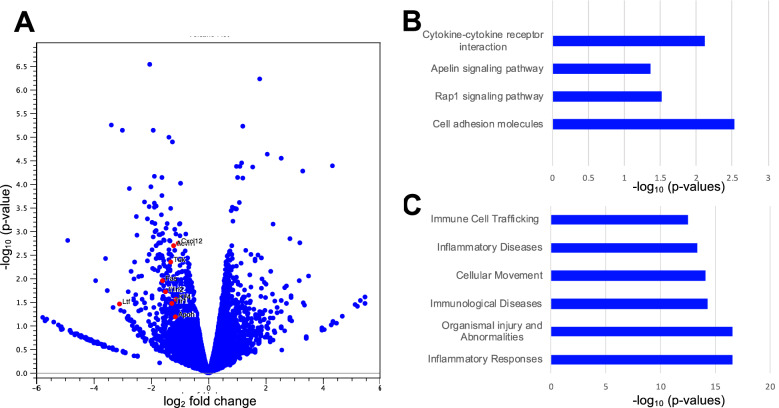
Sorbinil downregulates inflammatory-related signaling pathways one week after ONC. **(A)** The volcano plot shows differentially expressed (DE) genes associated with immune response that are downregulated in the whole retina one week after ONC (Sorbinil versus DMSO; *p-*value ≤ 0.1 and fold change is less than -2). **(B)** Pathway enrichment analysis using the KEGG database highlights the downregulation of various immune response-associated pathways one week after ONC with Sorbinil treatment. **(C)** The top 6 diseases and functions associated with the downregulated DE genes were determined by Ingenuity Pathway Analysis software. . N = 2 mice per Sorbinil group; N = 3 mice per DMSO group.

RNA sequencing was performed to explore the regulatory mechanism contributing to axonal degeneration delay after Sorbinil-dependent microglial inactivation. Microglial activation is associated with inflammatory and chemokine signaling during neuroinflammation^44^. As Sorbinil downregulates TNF-α expression and Iba1 upregulation after ONC (Fig. 2D,G), we reasoned that signaling pathways associated with inflammation could be downregulated after ONC, thereby delaying axon degeneration. We identified 438 differentially expressed (DE) genes, of which 302 genes were downregulated. The downregulated DE genes included cytokines TNF receptor- Fas and TGF-β receptor-ACVRL1, components of the TNF family and TGF-β family, respectively. Interferon regulatory factor genes interleukin 1 receptor-like 2 (IL1RL2) was also downregulated, suggesting downregulation of immune responses after Sorbinil treatment. IL1RL elicits an inflammatory response through MAPK signaling^45^. CXCL12, a known inflammatory chemokine^44^, was also downregulated by Sorbinil treatment. Other immune response associated downregulated genes were apolipoprotein H (APOH), lactoferrin (LTF), Kruppel-like factor 4 (KLF4), tunica interna endothelial cell kinase, a receptor tyrosine kinase (TEK), toll-like receptor 1 (TLR1), etc. (Fig. 4A). We further confirmed that Sorbinil treatment alone, in the absence of ONC, does not affect immune-related pathways (Supplementary Fig. 1). Thus, the downregulation of immune response genes is a direct result of Sorbinil’s neuroprotective and anti-inflammatory role. Pathway enrichment analysis depicts the downregulation of cytokine-cytokine receptor interaction, Repressor activator protein 1 (Rap-1), and Apelin signaling downregulation (Fig. 4B). Rap-1 and apelin are potential activators of inflammation through NF-kβ and P13k/Akt signaling pathways, respectively^46,47^. Additionally, ingenuity pathway analysis identified the downregulation of several genes involved in inflammatory response (Fig. 4C).

## Discussion

Chronic neuroinflammation is associated with various neurodegenerative diseases, including Alzheimer’s disease, Parkinson’s disease, and glaucoma. Modulating the signaling axis of the immune system may offer a potential therapeutic approach for treating neurodegenerative diseases^48^. As tissue insult increases, microglia reprogram into an activated proinflammatory state and play either a protective role in supporting cell survival^49^ or a harmful role in causing neuroinflammation^50^. Interestingly, neuroprotection was not observed in an animal microglia depletion model (using stimulating factor 1 receptor inhibitor PLX5622)^51^, which might be because deletion of microglia might eliminate both detrimental and neuroprotective effects in the retina. However, growing evidence shows that microglia modulation has been reported to inhibit disease progression^1,34,52^. Thus, we hypothesize that AR inhibition protects RGCs from death by suppressing microglia (or macrophage) activation. In addition, microglia also produce anti-inflammatory molecules and neurotrophic factors, which can repair a hostile CNS microenvironment after injury^53–56^. Here, we showed that AR inhibition suppresses pro-inflammatory cytokine secretion in RMG after ONC, an acute optic nerve injury model. Whether AR inhibition can also promote neuroprotective factors secretion in RMG remains unclear and would be an interesting direction in the future.

ON trauma and other CNS-related injuries are known to induce RMG activation^11,57,58^. RMG are associated with the activation of neuroinflammation^59^. AR polyol pathway causes NADH/NAD + redox imbalance leading to Sirt1-mediated inflammation^35^. Since Sirt-1 regulates NF-κB signaling pathway and it's downstream inflammatory responses^60^, it suggests that the production of cytokines, such as TNF-α, interleukins (IL)-1β, IL-6, IL-8, vascular cell adhesion molecule (VCAM-1) and intracellular adhesion molecule 1 (ICAM-1) could be attenuated by AR inhibition^61–63^. ONC triggers the activation of RMG along with macroglial cells like ASG and Müller glia^58^. We found that AR is highly expressed in RMG compared to ASG and RGC (Fig. 2A)^38^. AR is expressed in retinal vasculature including endothelium and pericytes, as well as in Müller glia^64^. It is possible that AR inhibition is also involved in other AR-expressing cell types, like Müller glia, that plays a possible neuronal protective role.

RMG-induced proinflammatory cytokines cause neuronal death in the retina^24^. Thus, we first studied if microglia activation by LPS-induced inflammatory cytokine expression can be attenuated by AR inhibition. TNF-α is one of the major cytokines released by RMG^65^. Our study corroborates previous reports that AR inhibition can attenuate TNF-α secretion in RMG (Fig. 1D)^38,63^. Additionally, Sorbinil has been previously reported to attenuate secretion of IL-1β in macrophages and activation of JNK and p38^66,67^. As Sorbinil does not affect BV2 cell viability, the attenuation of microglial distribution is unlikely caused by the cytotoxicity of Sorbinil (Fig. 1A). Furthermore, inflammation results in increased accumulation of ROS production which contributes to inflammatory cytokine secretion^68^. AR inhibition can alleviate oxidative stress by attenuating ROS and phagocytotic activity in microglial cells as recently reported by our group^67^. Published studies showed that activated microglia migrate into the inner nuclear or outer nuclear layer of the retina^9,69^, where the major neuronal cells reside. Therefore, it is important to inhibit activated RGC migration in the retina. We observed that AR inhibition suppressed LPS- and MCP-1-induced RMG migration (Fig. 1E and F). Taken together, our results suggest that AR inhibition can act as a promising therapy against ocular inflammation as it can prevent cytokine secretion and cell migration, thereby preventing damage to other retinal cells.

ONC causes resident retinal microglia activation and influx of blood-borne macrophages^70^. Although Iba1 staining does not distinguish between resident retinal microglia and blood-borne macrophages^71^, microglia are considered the main immune cells that respond to retinal degeneration^24,72^. Consistent with prior studies, we also found that ONC significantly recruits RMG to the ON and the retina^11^. In Western blot, we did not see the Iba-1 protein reduction in the Sorbinil-treated retina. However, we indeed observe fewer Iba-1 + cells in the Sorbinil treated retina. Additionally, we observe fewer Iba-1 + cells within the GCL of the retina after ONC suggesting that AR inhibition is involved in attenuating RMG migration (Fig. 2G). AR inhibition prevents cell migration, consistent with a role for AR inhibition in the prevention of RMG migration by suppressing cleaved MMP-9 formation^38^, the main enzymatic protein for cell migration.

Astrocyte glia (ASG) are also involved in ocular inflammation, especially in optic neuropathy^73^. Our data demonstrate that GFAP expression is increased by ONC and attenuated by Sorbinil but remains unchanged in the optic nerve. Because GFAP expression is primarily found in the optic nerve head region^73^ or the lesion of the crush site, we did not observe GFAP protein expression change in the optic nerve. As the AR level is relatively low in the ASG compared to RMG, we think AR inhibition should mainly target RMG and subsequently lead to secondary effects on ASG activation. The regulatory mechanism of AR between RMG and ASG is still unclear and remains an interesting future direction.

Finally, we want to know whether suppressing RMG activation could restore RGC loss and axon degeneration. Following standard protocol, we first observed the RGC and axon two weeks after ONC. However, we did not observe significant improvement in RGC survival, function, or axon regeneration. We next examined if AR inhibition played a role in delayed axon degeneration. Axonal degeneration one week after ONC results in RGC cell loss and distal axon terminal degeneration^74,75^. We found that AR inhibition promotes RGC survival and improves RGC function one week after ONC. In our study, we use CTB-555 and βIII-Tubulin protein expression to observe changes within ON axons one week after ONC. It should be noted that labeling axons with CTB-555 is an established method to evaluate axon regeneration and relies on axonal transport^76^. ONC results in damaged axonal transport within a few days after trauma^77^. Since we inject CTB-555 intravitreally two days before sample collection, it is possible that Sorbinil accelerates the CTB transport, leading to the elevation of CTB-555 + axon in ON one week after ONC. Therefore, we utilize βIII-Tubulin to assess the remaining axons one week after ONC, as this approach is an established and widely used surrogate marker for axonal integrity^43^. We observe that Sorbinil treatment plays a role in delaying distal axonal degeneration one week after ONC. Our results further corroborate a recent study showing that AR overexpression transgenic mice significantly lose their RGCs, which could be rescued by AR inhibition^78^.

RNA sequencing analysis revealed that cytokine and inflammatory associated genes are downregulated after AR inhibition (Fig. 4), which is consistent with prior studies showing that AR inhibition has been linked to TNF-α induced signaling^62,63,79^ and with our in vivo data of TNF-α downregulation (Fig. 2D). In addition, Fas, part of the TNF receptor superfamily, was downregulated by Sorbinil treatment. Inhibiting Fas signaling has been shown to provide neuroprotection in an inducible mouse model of glaucoma^80^. Fas promotes apoptosis through the Fas/caspase-8 pathway, which is the upstream signal of caspase-3^81,82^. Investigating whether Sorbinil reduces caspase-3 activation via suppressing Fas signaling and, as a result, delays axon degeneration will be an interesting future study. The RNA sequencing and our immunostaining data suggest that downregulation of TNF-induced signaling could be critical in improving RGC survival and delaying axon degeneration, although further studies are warranted. Downregulating inflammation through AR inhibition is not enough to promote axon regeneration. Thus, other processes along with suppressing inflammation must be involved to promote axon regeneration.

**Figure 5:**
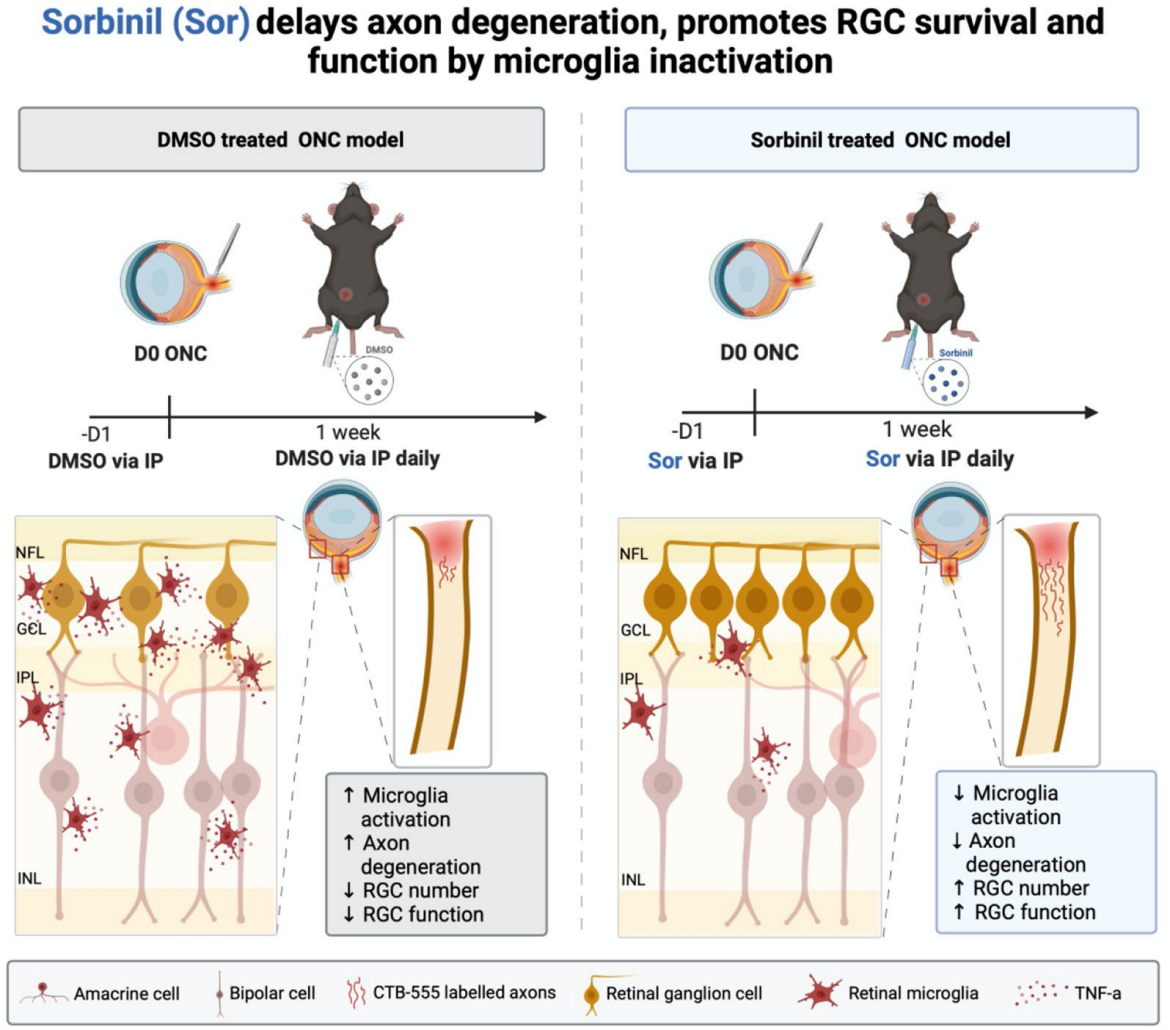
Model of AR-dependent regulation of microglia-RGC interaction. Optic nerve crush induces RMG distribution and TNF-α secretion in the retina and optic nerve, leading to RGC loss and axon degeneration. Daily treatment with Sorbinil for one week suppresses RMG infiltration and TNF-α secretion, which increases RGC survival and delays axon degeneration. The graphic was created with BioRender.com.

In summary, we report the first study showing that AR inhibition plays a transiently protective role in delaying RGC death and axon degeneration via anti-inflammatory responses (Fig. 5). AR inhibition alone is not effective in promoting axon regeneration. We thought that AR inhibition attenuates pro-inflammatory cytokines secretion and as a result, delays axonal degeneration in a short period. However, it cannot stop injured axons from dying in a long run. Growing studies of gene therapy show enhancing RGC survival and long-distance axon regeneration^12,83–85^, however, the functional restoration in the visual circuit remains a challenge. For example, a combinational strategy using AR inhibitor and shKLF9/Sox11, known axon regeneration promotors, might provide synergistic effects on axon regeneration. Furthermore, in this study we used ONC as an acute model for RGC injury. It will be an exciting future study to confirm if Sorbinil also plays a protective role in different RGC injury models like intraocular pressure (IOP) and the reperfusion model. Our results are encouraging as we can combine AR inhibition with gene therapy to develop a novel therapeutic strategy, hoping to repair vision impairment in optic neuropathies.

## Methods

### Animals

All animal experiments were performed in accordance with ARVO and ARRIVE guidelines for the Use of Animals in Research, and approved by the University of Pittsburgh School of Medicine Institutional Animal Care and Use Committee (IACUC; protocol# 21018446). Both sexes of C57BL/6 mice were used for this study.

### MTT cell viability assay

The toxicity effect of Sorbinil on BV2 cell viability was determined using 3-(4,5-Dimethyl-2-thiazolyl)-2,5-diphenyl-2H-tetrazolium bromide (MTT) (Tocris, #5224). Briefly, BV2 (3000 cells/well) were seeded in a 96-well plate in DMEM/F12 + 10%FBS + 1%PS and incubated overnight at 37 °C, 5% CO2. The glucose concentration in our BV2 medium is 17 mM. The cells were treated with increasing concentrations of Sorbinil 5 μM, 10 μM, 20 μM and 40 μM overnight at 37 °C, 5% CO2. 5 mg/ml of MTT were added to each well for 4 h. Levels of MTT were determined by measuring the absorbance at 570 nm.

### D-sorbitol colorimetric assay

BV2 (5 × 10^4^ cell/well) were seeded in a 6-well plate overnight at 37 °C, 5% CO2 DMEM/F12 (#11330032, ThermoFisher) + 10%FBS + 1%PS. The glucose concentration in our BV2 medium is 17 mM. BV2 cells were treated with Sorbinil (10 μM) and DMSO control for three days. Subsequently, cells were prepared to measure D-Sorbitol concentration using the D-Sorbitol Colorimetric Assay Kit (#MAK010, Sigma-Aldrich) as per manufacturer protocol. Levels of D-Sorbitol were determined by measuring the absorbance at 560 nm.

### ELISA assay

BV2 (10^3^ cells) were incubated in a 24-well plate, and media were collected after lipopolysaccharide (LPS) exposure. Mouse tumor necrosis factor (TNF)-α DuoSet ELISA Development kit (R&D Systems, Inc., Minneapolis, MN, USA) was used to measure TNF-α secreted in the media. The optical density was detected using a microplate reader. A standard curve was generated and used to collect the cytokine level measurement.

### High-throughput single-cell migration assay by microfluidics

The cell migration assay of BV2 microglia was performed using a microfluidic single-cell migration platform modified from previous work^86,87^. The migration channel is designed to be 5 μm in height and 10 μm in width. The device was primed with a collagen solution (1.45 mL Collagen Type 1, (354236, BD Biosciences) and 0.1 mL acetic acid in 50 mL DI Water)) to enhance cell adhesion. Right before the migration experiment, the devices were rinsed with cell culture media. Cells were first stained by green CellTracker dye (ThermoFisher C2925) following the manufacturer’s protocol and then trypsinized, centrifuged, and re-suspended to a concentration of 4 × 10^5^ cells/mL for loading onto the devices. After cell loading, the devices were incubated for 1 h to enhance cell adhesion. BV2 microglia were pretreated with Sorbinil (20 μM, Sigma-Aldrich) for 1 h. LPS (100 ng/mL, Sigma-Aldrich) and MCP-1 chemo-attraction (30 ng/mL, Peprotech) were used to stimulate and induce cell migration. Sorbinil was used to inhibit cell motility. The microfluidic devices were incubated for 18 h, and the migration distance was measured based on the final cell position. The images were analyzed by a custom MATLAB code^87,88^. Cells were identified based on their fluorescence, and debris was ignored by their small size. In this work, 3–6 independent replicates (~ 300–600 migration channels) were performed. The Violin graphs were plotted using Prism (v.9.3). As cell migration results do not follow a normal distribution, a non-parametric Mann–Whitney U test was used for comparisons of cell motility with a significance level of 0.05 considered statistically significant.

### Cell cultures

Culture of primary mouse RGC, ASG, and RMG was performed as previously described^12,89^. Briefly, to isolate RGCs, mouse retinas from postnatal P2 pups were dissociated with papain (Worthington Biochemical Corp). Macrophages and endothelial cells were removed by immunopanning using an anti-macrophage (CD68) antibody. RGCs were further purified via immunopanning using CD90.1. Purified RGCs were cultured on poly-D-lysine (PDL)/laminin-coated plates in full Sato medium as previously described^12^.

Mouse ASG were purified from postnatal P1-P4 pups as reported^89^. Cortices without meninges were collected and dissociated to achieve a single cell suspension. Cells were plated on a PDL coated T-75 flask in DMEM containing 10% fetal bovine serum (FBS) and penicillin/streptomycin. After the cells reached confluency, the flask was shaken at 180 rpm for 60 min to detach the microglia. Fresh media was added, and subsequently, the flask was shaken at 240 rpm for six hours to separate oligodendrocyte precursor cells. The remaining astrocyte layer was then cultured for western blot sample collection.

RMG were isolated and dissociated from the retinas of 8–10-week-old C57BL/6 mice^38^. Dissociated cells were cultured for four weeks in DMEM containing 4 mM L-glutamine, 10% FBS, and penicillin/streptomycin. Once cells were confluent, the flask was shaken at 100 rpm on an orbital shaker for 1 h. Detached cells were grown to collect primary mouse RMG for western blotting.

### Western blot

Laemmli sample buffer (Sigma-Aldrich) was used to collect protein samples. Lysates were heated to 100 °C for 10 min. Samples were loaded onto precast SDS-PAGE gels (Bio-Rad) and run until completion. Proteins were transferred to a polyvinylidene difluoride membrane (PVDF) using a semi-dry blotter (Bio-Rad). Membranes were blocked at room temperature for 1 h using LI-COR “Intercept” Blocking Buffer. Blots were incubated with primary antibody at 4 °C overnight in LI-COR “Intercept” Antibody Buffer for immunodetection. Primary antibodies are mouse anti-AR (1:1000; #sc-166918; Santa Cruz Biotechnology Inc., Dallas, TX), rabbit anti-Iba1 antibody (1:1000; #019-19741; Wako), rabbit anti-RBPMS primary antibody (1:500; #1830; PhosphoSolution), rabbit p-p38 (1:500; #4511S; Cell signaling Technology, Inc.), p38 (1:1000; #8690S; Cell signaling Technology, Inc.), GFAP (1:1000; #6370S; Cell signaling Technology, Inc.),)and GAPDH (1:1000; #2118S; Cell signaling Technology, Inc). The membrane was incubated with species-specific secondary antibodies linked to near IR dyes (LI-COR, IRDye 680RD donkey anti-mouse (#926-68072), IRDye 800CW Goat anti-rabbit (#926-3211) at 1:10,000 dilution for 4 h at room temperature, washed, and imaged on a LI-COR Odyssey IR in a linear range.

### Immunostaining

For immunostaining, sections were incubated in blocking buffer (5% normal goat serum and 0.1% Triton X-100 in PBS) for 1 h at room temperature. After washing with PBS three times, sections were incubated with the primary antibodies overnight at 4 °C and with the secondary antibodies for 4 h at room temperature. The primary antibodies used for staining were: rabbit anti-Iba1 antibody (1:200; Wako, Richmond, VA, USA), and cleaved caspase-3 (1:200; #9661S, Cell Signaling Technology, Danvers, MA). After incubation at 4 °C overnight, the cells were stained with Alexa Fluor® 488 Goat Anti-rabbit (#A11034) and 4′,6-diamidino-2-phenylindole (DAPI) (1:5000, (#D9542), Sigma-Aldrich) for 1 h. Cleaved caspase-3 + cells within the GCL layer were counted for apoptotic cell staining. DAPI was used to mark the GCL layer and used to identify condensed nuclei. At least 5 retinal sections were counted per retina sample to give an overall landscape of cleaved caspase-3 positive cells per retina. Images were obtained using an Olympus Life Science IX83 Inverted Microscope.

### Optic nerve crush

The procedures of ONC were followed as previously described^76^. Briefly, 8- to 10-week-old mice were anesthetized with ketamine/xylazine. The mice were preinjected with Sorbinil (10 mg/kg) or DMSO intraperitoneally for 24 h before ONC. ON of the left eye (OS) was exposed from the outer canthus and pinched for 5 s with a Dumont Fine Science Tools #5 self-closing forceps ∼1.5 mm behind the globe. The right eye (OD) was left uninjured to act as a control. Animals were treated with Sorbinil or DMSO daily for the experiment duration. ONs were collected one week and two weeks after ONC. Two microliters of cholera toxin subunit B (CTB)-conjugated Alexa Fluor 555 (CTB-555, 2 μg/μl; Invitrogen, (#C22843)) was intravitreally injected as an anterograde tracer to visualize axons two days before euthanization. Animals were perfused with 4% Paraformaldehyde (PFA) before ON and retinas collection.

### Quantitative real time PCR

Total RNA was isolated from retinal tissues (control + treatment retinas) collected 1 week after ONC. RNeasy Microarray Tissue MiniKit, QIAGEN was used to perform RNA extraction according to the manufacturer’s protocol. RNA (500 ng) was reverse transcribed using iScript cDNA Synthesis Kit, Bio-Rad. qPCR was performed using iTaq Universal SYBRGreen Supermix, Bio-Rad according to the manufacturer’s protocol. SYBR green primers- CD68, Il-6, Bcl-2, and GAPDH were purchased from Integrated DNA Technologies. Experiments were performed in triplicates.

### RGC survival analysis

RGC survival analysis was performed according to the previously established protocol^12^. Briefly, retinas were dissected and fixed in 4% PFA for 1 h, permeabilized with 3% Triton X-100 (Sigma-Aldrich) and 1.5% Tween 20 (Sigma-Aldrich) for 1 h, blocked with 10% normal goat serum (NGS) in PBS for 1 h, and then incubated with a rabbit polyclonal anti-RBPMS primary antibody (1:500; (#1830), PhosphoSolution) overnight at 4 °C. Flatmount retina samples were washed three times, ten minutes each, with PBS and incubated with Alexa Fluor® 647- Goat Anti-rabbit (#A21244) (1:500; Life Technologies) overnight. The explants were then washed twice, ten minutes each, stained with DAPI (1:5000 in PBS) for 15 min, washed twice for 10 min each, and sealed under 1.5-mm coverslips with anti-fade mounting medium (ProLong Gold, Life Technologies) before imaging via fluorescence microscopy (Zeiss). Each retina was divided into four quadrants, and one digital micrograph was taken randomly from each of the four peripheral areas 3 mm from the ON head. RBPMS-positive cells were counted manually in a masked fashion and presented as cells per millimeter squared.

### Axon number counting

ON were dissected, fixed in PFA for 1 h at room temperature, and subsequently washed in PBS. ON were then incubated in 15% sucrose at 4 °C overnight and 30% sucrose at 4 °C overnight before mounting in Optimal Cutting Temperature mounting medium (Thermo Fisher Scientific). 10-μm-thick cryosections were cut for both ON and retina. ON sections were imaged and analyzed as previously described^76^. The number of CTB + axons within every 250 μm from the crush site was manually counted to the end of the longest regenerating axons. Total CTB + axons per ON were calculated using the formula previously described^90^.

### Axon degeneration analysis

βIII-Tubulin staining was performed on the injured ON of the mouse treated with DMSO or Sorbinil one week after ONC to determine axonal integrity. βIII-Tubulin staining helps visualize microtubules that break down early in Wallerian degeneration^43^. For immunostaining, sections underwent the previously described method (Sect. 2.6.). Primary antibody used was βIII-Tubulin (1:500; #5568S, Cell Signaling Technology, Danvers, MA), and the secondary antibody used was Alexa Fluor® 488 Goat Anti-rabbit (#A11034).

To measure βIII-Tubulin density, ON were imaged at the same intensity and 10X magnification on Olympus Life Science IX83 Inverted Microscope. Density was measured using a fixed area of 500 μm width and 200 μm height immediately after the crush site using Fiji^91^. ONs from four mice per group were analyzed.

### Pattern electroretinography (PERG)

Pattern electroretinography (PERG) analysis was performed according to a previously established protocol^92^. Briefly, PERGs were measured in adult mice using the Celeris ERG stimulator (Diagnosys) per the manufacturer’s instructions. Mice were anesthetized, and their eyes were dilated before placing a pattern ERG electrode on the corneal surface. A visual stimulus was aligned along with the mice’s pupil that generated black and white alternating contrast-reversing bars. Pattern ERGs were measured between a peak and adjacent trough of the waveform. Four mice per group and 300 reads per eye were recorded.

### RNA sequencing

Seven days after ONC, the injured retina of the mouse treated with DMSO or Sorbinil was dissected, and total RNA was extracted. This sample preparation was conducted in triplicates for both conditions. Sample preparation, library preparation, and quality control analysis were conducted by the Health Sciences Sequencing Core at Children’s Hospital of Pittsburgh. RNA quality was verified using Agilent Tapestation RNA and Qubit Fluorometric Quantification. Library preparation was conducted with the TruSeq Total RNA library kit (Illumina) using at least 1 μg of total RNA following the manufacturer’s instruction. Using NextSeq500 (Illumina), libraries were sequenced with 40 M reads per sample.

FASTQ files were analyzed using CLC Genomics Workbench v22 (QIAGEN Digital Insights). Raw sequencing reads were imported into CLC Genomics Workbench for RNA sequencing data analysis and computing differentially expressed (DE) genes between Sorbinil vs. DMSO after ONC. Quality check was performed on the imported reads. Adaptor sequences were trimmed, and reads were aligned to the GRCm39/mm39 version of the mouse reference genome using default settings. Quality check was performed on the mapped reads. Principal component analysis (PCA) of mapped reads led to the exclusion of an individual replicate from the Sorbinil group. Differential expression of genes between Sorbinil treatment vs. DMSO controls was determined if the *p-*value ≤ 0.1 and fold change is less than -2. Adjusted *p-*value was not used to compute DE genes^93^. Volcano plots were generated using CLC Genomic Workbench v22. Pathway enrichment analyses were performed on NIH Database for Annotation, Visualization, and Integrated Discovery (DAVID) 2021 (https://david.ncifcrf.gov) using the KEGG database. The Ingenuity Pathway Analysis (IPA) (QIAGEN Digital Insights) tool was also used on DE genes to identify statistically enriched biological pathways and functions. The RNA-Seq data is available at the Gene Expression Omnibus Web site (http://www.ncbi.nlm.nih.gov/geo/) under accession GSE200233.

### Statistical analysis

Results are shown as the mean ± SEM of at least three experiments. The exact number of mice used per experiment is highlighted in the figure legend. Data were analyzed using one-way ANOVA and post hoc t-test with Tukey correction and/or an unpaired t-test with *p *< 0.05 considered significant. Graphs were constructed using Prism 9 (GraphPad, La Jolla, CA).

## Supplementary Information

Supplementary Figure S1.

Supplementary Figure S2.

## Data Availability

The datasets generated and analyzed in this study are available upon request. Please contact the corresponding author.
