# Supplementary material for: Aldose reductase inhibition decelerates optic nerve degeneration by alleviating retinal microglia activation
Source: Sci Rep. 2023 Apr 5;13:5592. doi: 10.1038/s41598-023-32702-5 (PMC10076364; doi:10.1038/s41598-023-32702-5)
Supplement: Supplementary file 1 — Supplementary Figure S1. [file 41598_2023_32702_MOESM1_ESM.pdf]

A

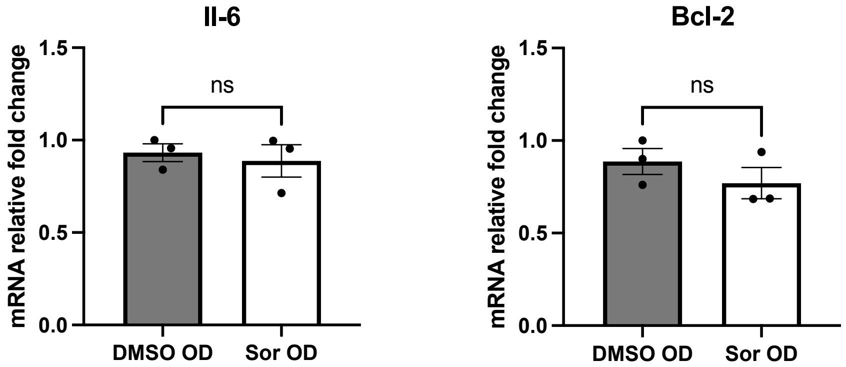

Figure S1. Sorbinil treatment, in the absence of ONC, does not cause changes to inflammation related genes. (A) Fold change expression of *il-6* between DMSO and Sorbinil treated retina 1 week after treatment. **(B)** Fold change expression of *bcl-2* between DMSO and Sorbinil treated retina 1 week after treatment.
