# Supplementary material for: Aldose reductase inhibition decelerates optic nerve degeneration by alleviating retinal microglia activation
Source: Sci Rep. 2023 Apr 5;13:5592. doi: 10.1038/s41598-023-32702-5 (PMC10076364; doi:10.1038/s41598-023-32702-5)
Supplement: Supplementary file 2 — Supplementary Figure S2. [file 41598_2023_32702_MOESM2_ESM.pdf]

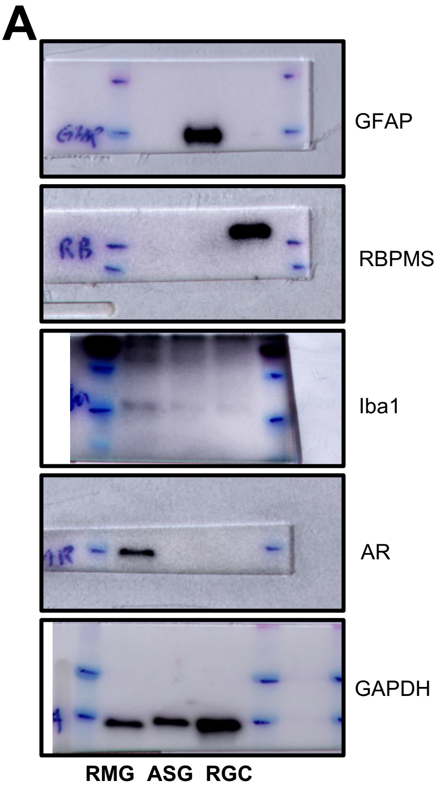

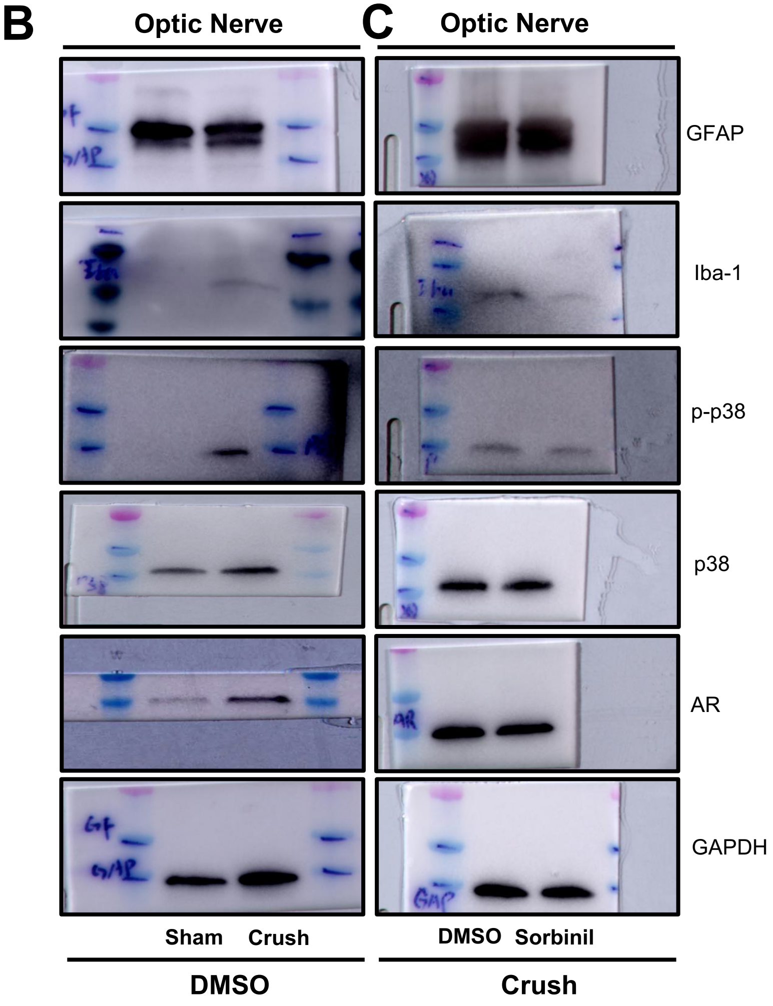

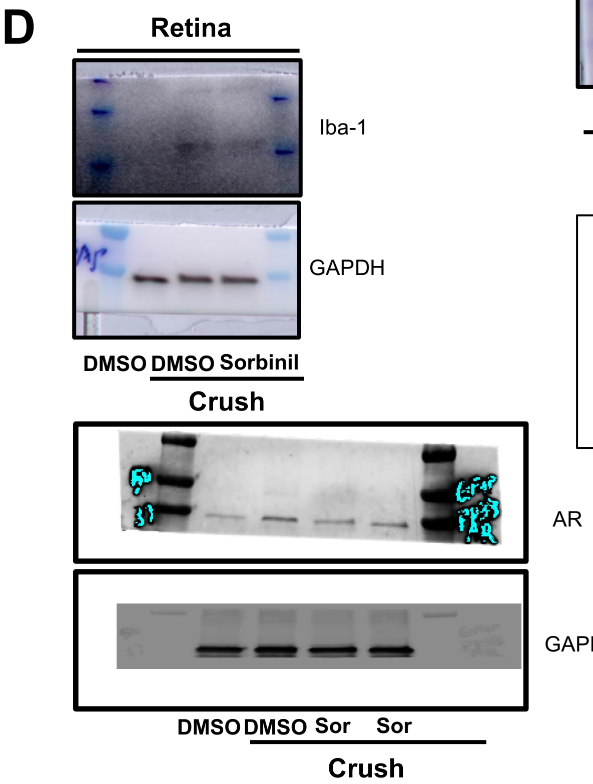

Figure S1. Full length uncropped Western blots images. Membranes were cut to allow blotting for multiple antibodies. (A) Full length image for figure 2A. **(B)** Full length image for figure 2B. **(C)** Full length image for figure 2C. **(D)** Full length image for figure 2E.
